# Genetic analysis of growth traits in Harnali sheep

**DOI:** 10.14202/vetworld.2016.128-132

**Published:** 2016-02-08

**Authors:** Lalit, Z. S. Malik, D. S. Dalal, S. P. Dahiya, C. S. Patil, Ravinder Dahiya

**Affiliations:** 1 Department of Animal Genetics and Breeding, Lala Lajpat Rai University of Veterinary and Animal Sciences, Hisar, Haryana, India; 2 Department of Animal Nutrition, Lala Lajpat Rai University of Veterinary and Animal Sciences, Hisar, Haryana, India

**Keywords:** correlations, growth traits, Harnali sheep, heritability

## Abstract

**Aim:**

The present investigation was to study genetic characteristics of Harnali sheep with respect to growth performance and to estimate genetic parameters.

**Materials and Methods:**

The 22 years (1992-2013) data of growth traits of a 1603 synthetic population of Harnali sheep maintained at Lala Lajpat Rai University of Veterinary and Animal Sciences, Hisar, was utilized for this study. A mixed methodology with regression on their dam's weight was used to study the effect of non-genetic factors on growth traits. Heritability, genetic and phenotypic correlations were estimated using paternal half-sib analysis for body weight at various ages and average daily gain (ADG) for different growth periods.

**Result:**

The overall least squares mean of body weights recorded for birth weight (BW), weaning weight (WW), six months body weight (SMW), one yearling body weight (YBW), average daily gain from birth to 3 months (ADG1) and average daily gain from 3 to 12 months (ADG2) were 3.35±0.05 kg, 12.41±0.08 kg, 16.30±0.12 kg, 21.88±0.08 kg, 100.66±0.86 g/day and 35.07±0.39 g/day, respectively. The effects of year of birth significantly (p<0.01) influenced the BW, WW, SMW, YWB, ADG1 and ADG2. The effects of sex of lamb significantly (p<0.01) influenced the BW, WW SMW, YWB, ADG1 and ADG2. The effects of dam's weight at lambing significantly (p<0.01) influenced BW, WW, SMW, YWB, ADG1 and ADG2. No definite trend was observed over the years for the averages of body weight and gain. The heritability estimates of BW, WW, SMW, YBW, ADG1 and ADG2 were 0.40±0.05, 0.38±0.05, 0.45±0.06, 0.29±0.05, 0.40±0.06 and 0.33±0.02, respectively. The male lambs were significantly heavier than females at all stages of growth. The heritability estimates were moderate for all the growth traits and high genetic correlations of BW and WW with SMW were found.

**Conclusion:**

Due to high heritability and positive correlations of SMW with other body weights and daily gain, it was concluded that selection on the basis of SMW would be the best approach to improve growth performance in Harnali sheep.

## Introduction

Sheep is one of the important species of livestock in India. They contribute greatly to the agrarian economy, especially in the livelihood of a large proportion of small and marginal farmers and landless laborers. The sheep population in India is estimated to be about 65.07 million with second ranking in the world [1]. There are 40 descript breeds of sheep distributed in various agroclimatic zones of the country [2]. A cross breeding of indigenous sheep with exotic breeds has been in practice since long to bring about the improvement in both wool and mutton production. Such attempts have been resulted in the evolution of some superior breeds, *viz*., Hissardale, Kashmir Merino, Bharat Merino, etc.

The growth rate is an economic trait of interest in domestic animals as growth of the lambs is a reflection of the adaptability and economic viability of the animal and hence may be used as criteria for the selection among breeds and the individual within breeds [3]. Fast growth rate ultimately determines their meat producing capability up to marketing age. The study of body weights also helps or even guides the breeders to determine the optimum managemental practices so as to maintain the gain at an optimum level. Harnali sheep is a three breed cross by 37.5% Nali and 62.5% exotic inheritance (Merino and Corriedale with equal inheritance, i.e., 31.25) developed at Lala Lajpat Rai University of Veterinary and Animal Sciences, Hisar for superior wool production. At present about 300 animals of Harnali sheep are maintained at university farm besides several animals have been disseminated to farmers around Hisar. The literature is dotted with conflicting and sporadic reports regarding genetic parameters of growth traits in sheep [3-6].

Therefore, the present investigation was aimed to estimate the genetic parameters of growth traits in Harnali sheep.

## Materials and Methods

### Ethical approval

All the procedures have been conducted in accordance with the guidelines laid down by the Institutional Ethics Committee.

### Data recorded and generated

This study was conducted on the data collected over a period of 22-year (1992-2013) pertaining to growth trait records of 1603 Harnali sheep maintained at Lala Lajpat Rai University of Veterinary and Animal Sciences, Hisar. Hisar is located at 29°09’N, 75°42’E, altitude 215 m with average rainfall 490.6 mm and average temperature ranges between 17.6 and 32.5°C. The traits included in the analysis were birth weight (BW), weaning weight (WW), six months body weight (SMW), one yearling body weight (YBW), average daily gain from birth to 3 months (ADG1), average daily gain from 3 to 12 months (ADG2). The lambs were allowed to be suckling up to 90 days. They were also provided with concentrate feed after 2 months of age.

### Mixed linear model with regression on dam's weight

The effect of non-genetic factors *viz*. year, sex and dam's weight at lambing on various traits were studied by least square analysis technique using the following mixed model, Y_ijkl_ = μ + S_i_ + Y_j_ + S_k_ + b (X_ijkl_− X̄) + e_ijkl,_ Where, Y_ijkl_ is observation on l^th^ lamb belonging to i^th^ sire (i=175) born in j^th^ year to k^th^ sex; μ is the overall mean; S_i_ is the random effect of i^th^ sire, Y_j_ is fixed effect of j^th^ year (j=1-22), S_k_ is the fixed effect of k^th^ sex (k=1, 2); b is the partial regression of traits on dam's weight at lambing; X_ijkl_ is the dam's weight corresponding to Y_ijkl_; X– is the mean dam's weight at lambing; e_ijkl_ is the random error associated with each observation and assumed to be normality and independently distributed with mean zero and variance σ^2^_e_.

### Statistical analysis

The least squares and maximum likelihood computer program [7] was used to estimate the effect of various factors on different growth traits. Modified Duncan's multiple range test [8] was used for comparing subgroup means. Heritability estimates for different growth traits were obtained from sire component of variances using paternal half-sib correlation method [9]. The standard errors of heritability estimates were obtained using the formula given by Swiger *et al*. [10]. Genetic correlations among different traits were calculated from sire components of variances and co-variances. The standard errors of genetic correlations were estimated using the formula given by Robertson [11]. Phenotypic correlations among various traits were calculated from total variances and covariances. The standard error of phenotypic correlation was computed using the formula given by Snedecor and Cocharan [12].

## Results and Discussion

### Least squares analysis of body weights

The analysis of variance and least squares mean along with standard errors to identify the effect of non-genetic factors on the observed body weights recorded at BW, WW, SMB and YBW of age are given in Tables-1 and 2, respectively. The overall mean for BW, WW, SMW and YBW were 3.35±0.05 kg, 12.41±0.08 kg, 16.30±0.12 and 21.88±0.08 kg, respectively. The overall least squares mean of body weights recorded for BW, WW, SMW, YBW, ADG1 and ADG2 were 3.35±0.05 kg, 12.41±0.08 kg, 16.30±0.12 kg, 21.88±0.08 kg, 100.66±0.86 g/day, 35.07±0.39 g/day, respectively. This is in close agreement with earlier findings in the same breed [13]. The averages were, however, higher than those reported as 1.82±0.03 kg in Garole × Malpura crossbred [14], 3.25±0.17 kg in synthetic sheep [15] for BW; in Nali crosses for WW, SMW and YBW [3,13,15]. Higher WW in lamb reflects better mothering ability of the dam's as compared to other breeds.

**Table-1:** Analysis of variance for growth traits.

Sources	Degree of freedom	Mean squares					

		BW	WW	SMW	YBW	ADG1	ADG2
Sire	174	0.17	5.46	10.90	8.68	632.26	153.60
Year	21	0.92**	27.40**	19.37**	22.39**	3236.87**	407.95**
Sex	1	0.59*	31.06**	106.42**	317.41**	2850.46*	2056.05**
Dam’s weight at lambing (linear regression)	1	100.62**	868.64**	1668.39**	1853.48**	46664.65**	2529.51**
Error	1405	0.13	4.12	7.34	8.30	489.95	134.65

* Significant at p<0.05

** Significant at p<0.01, BW=Birth weight, WW=Weaning weight, SMW=Six months body weight, YBW=One yearling body weight, ADG1=Average daily gain from birth to 3 months, ADG2=Average daily gain from 3 to 12 months

**Table-2:** Least squares means along with standard errors for growth traits.

Effects	Number of observation	Traits					

		BW (kg)	WW (kg)	SMW (kg)	YBW (kg)	ADG1 (g)	ADG2 (g)
Overall (μ)	1603	3.35±0.05	12.41±0.08	16.30±0.12	21.88±0.08	100.66±0.86	35.07±0.39
Year							
1992	55	3.62^a^±0.19	11.72^bc^±1.67	15.28^b^±1.42	18.66^b^±1.51	89.98^b^±11.64	25.70^bc^±6.01
1993	84	3.64^a^±0.18	11.67^bc^±1.02	15.58^b^±1.37	19.57^b^±1.45	89.25^b^±11.16	29.24^b^±5.85
1994	22	3.69^a^±0.19	11.82^bc^±1.05	14.40^b^±1.40	19.95^b^±1.49	90.40^b^±11.44	30.09^b^±5.99
1995	83	3.59^a^±0.14	12.00^b^±0.78	16.60^ab^±1.05	20.29^b^±1.11	93.45^b^±8.54	30.71^b^±4.47
1996	92	3.63^a^±0.12	11.43^bc^±0.68	17.62^a^±0.91	20.94^ab^±0.97	86.61^bc^±7.44	35.24^ab^±3.90
1997	77	3.43^a^±0.13	09.52^c^±0.75	16.23^ab^±1.00	20.34^b^±1.06	67.61^c^±8.19	40.07^a^±4.29
1998	38	3.21^ab^±0.13	11.33^bc^±0.72	17.15^a^±0.96	19.48^b^±1.02	90.25^b^±7.84	30.18^b^±4.10
1999	75	3.34^ab^±0.12	10.44^bc^±0.66	16.39^ab^±0.89	19.82^b^±0.94	78.87^bc^±7.24	34.73^ab^±3.79
2000	90	3.30^ab^±0.11	11.38^bc^±0.59	17.19^a^±0.79	20.76^ab^±0.84	89.74^b^±6.47	34.75^ab^±3.38
2001	56	3.44^a^±0.11	10.88^bc^±0.63	16.50^ab^±0.84	22.63^ab^±0.89	82.61^bc^±6.83	43.52^a^±3.57
2002	65	3.30^ab^±0.11	12.41^b^±0.59	18.00^a^±0.79	21.49^ab^±0.83	101.22^b^±6.43	33.60^ab^±3.37
2003	108	3.29^ab^±0.10	11.57^bc^±0.54	16.36^ab^±0.72	23.02^a^±0.76	92.03^bc^±5.87	42.42^a^±3.07
2004	95	3.14^b^±0.10	11.08^bc^±0.56	16.42^ab^±0.75	22.62^ab^±0.80	88.26^bc^±6.15	42.71^a^±3.21
2005	49	2.91^b^±0.11	10.36^bc^±0.61	14.18^b^±0.81	21.56^ab^±0.86	82.73^bc^±6.64	41.48^a^±3.47
2006	85	3.35^ab^±0.10	11.98^bc^±0.58	15.26^b^±0.78	23.12^a^±0.83	95.90^b^±6.37	41.25^a^±3.33
2007	97	3.61^a^±0.11	12.82^b^±0.59	16.01^ab^±0.79	22.60^ab^±0.84	102.33^b^±6.48	36.21^ab^±3.39
2008	100	3.32^ab^±0.11	14.39^ab^±0.61	16.52^ab^±0.82	24.33^a^±0.87	123.07^ab^±6.68	36.80^ab^±3.49
2009	81	3.17^b^±0.15	14.15^ab^±0.86	16.35^ab^±1.14	24.43^a^±1.21	122.01^ab^±9.32	38.06^a^±4.88
2010	96	3.27^ab^±0.15	14.31^ab^±0.83	16.22^ab^±1.11	25.06^a^±1.18	122.67^ab^±9.09	39.83^a^±4.76
2011	70	2.99^b^±0.16	14.89^a^±0.88	15.76^ab^±1.17	23.95^a^±1.25	132.19^a^±9.62	33.54^ab^±5.04
2012	70	3.14^b^±0.18	15.35^a^±1.01	17.55^a^±1.35	23.56^a^±1.43	135.69^a^±11.02	30.41^b^±5.77
2013	15	3.30^ab^±0.31	15.89^a^±1.72	17.96^a^±2.31	23.13^a^±2.45	157.67^a^±18.85	20.92^c^±9.88
Sex							
Female	862	3.33^b^±0.02	12.26^b^±0.09	16.02^b^±0.14	21.40^b^±0.11	99.22^b^±1.01	33.84^b^±0.48
Male	741	3.37^a^±0.02	12.56^a^±0.10	16.58^a^±0.14	22.36^a^±0.12	102.10^a^±1.09	36.29^a^±0.52
Regression Dam’s weight at lambing		0.078±0.003	0.23±0.016	0.32±0.02	0.33±0.02	1.68±0.171	0.39±0.09

Means with different superscript for an effect differed significantly (p<0.05). BW=Birth weight, WW=Weaning weight, SMW=Six months body weight, YBW=One yearling body weight, ADG1=Average daily gain from birth to 3 months, ADG2=Average daily gain from 3 to 12 months

The effects of year of birth significantly (p<0.01) influenced the BW, WW, SMW, YWB, ADG1 and ADG2. The effects of sex of lamb significantly (p<0.01) influenced the BW, WW SMW, YWB, ADG1 and ADG2. The effects of dam's weight at lambing significantly (p<0.01) influenced BW, WW, SMW, YWB, ADG1 and ADG2 (Table-1). These results are similar to the findings of earlier researcher [3,16]. Various researchers also reported significant effect of year of birth and sex of lamb on WW and SMW [17-20]. Male lambs were heavier than female for the body weight at all stages. A significant effect of sex on WW and SMW has also been reported in Deccani sheep [21]. The effect of weight of dam at lambing showed an increasing trend in all age groups which may be due to mothering ability and milk yield. Heavier dams gave birth to heavier lambs because of better nutrition and more uterine space provided by them for developing fetus [22]. The results are in conformity with the findings of Dey [23] and Sehrawat [13] in crossbred sheep, Balasubramanyam and Kumarasamy [24] and Devendran *et al*. [20] in Madras Red sheep. Variation in environmental conditions, feed and fodder availability prevailing in different years could lead to significant year differences.

### ADG

The analysis of variance and least squares means along with standard errors to identify the effect of non-genetic factors on the average daily weight gain is given in Tables-1 and 2, respectively. The overall average daily weight gains during birth to 3 months (ADG1) and 3-12 months (ADG2) were 100.66±0.86 and 35.07±0.39, respectively, growth during birth to 3-month period is rapid compared to 3-12 months indicating that the culling of the lambs for feedlot purpose should be done at early ages to increase the economic returns. The effects of year of birth significantly (p<0.01) influenced the ADG1 and ADG2. The effects of sex of lamb significantly (p<0.01) influenced the ADG1 and ADG2. The effects of dam's weight at lambing significantly (p<0.01) influenced on ADG1 and ADG2. A higher pre-weaning and post-weaning daily gain were also found in male lambs than females. These findings are in agreement with Dey [23], Sehrawat [13], Prince *et al*. [22], and Ganeshan *et al*. [25]. Significant variations found over the years could be due to the different management conditions and maternal environment experienced by the lambs.

### Genetic parameters

#### Heritability estimates

The estimates of heritability along with standard errors for BW, WW, SMW, YBW, ADG1 and ADG2 are given in Table-3. The estimates of heritability for SMW was 0.45 indicating a high degree of genetic variability in this trait. The heritability estimates of BW, WW, SMW, YBW, ADG1 and ADG2 were 0.40±0.05, 0.38±0.05, 0.45±0.06, 0.29±0.05, 0.40±0.06 and 0.33±0.02, respectively, suggesting that there is the considerable scope of improvement in these traits by mass selection. Similar results for these traits were also reported by Baneh *et al*. [26], Gowane *et al*. [14] and Vivekanand *et al*. [27]. At 6 months, maternal effects are reduced considerably and there is also similar plane of nutrition for all the individuals in the flock. This might has helped to reduce the environmental variability resulting in higher heritability values. Therefore, weight at 6 months can be considered a good criterion for selecting animals.

**Table-3:** Estimates of heritability (diagonal), genetic (above diagonal) and phenotypic (below diagonal) correlations along with standard errors among growth traits.

Traits	BW	WW	SMW	YBW	ADG1	ADG2
BW	0.40±0.05	0.30±0.11	0.59±0.10	0.26±0.12	0.34±0.22	−0.10±0.12
WW	0.21**±0.02	0.38±0.05	0.54±0.10	0.31±0.12	0.99±0.01	0.55±0.13
SMW	0.47**±0.02	0.46**±0.02	0.45±0.06	0.45±0.10	0.29±0.11	0.42±0.10
YBW	0.25**±0.02	0.22**±0.02	0.39**±0.02	0.29±0.05	0.17±0.12	0.69±0.07
ADG1	0.29**±0.02	0.98**±0.01	0.42**±0.02	0.18**±0.02	0.40±0.06	0.49±0.30
ADG2	0.09**±0.02	−0.45**±0.02	0.68**±0.01	0.80**±0.01	−0.48**±0.02	0.33±0.02

** Significant at p<0.01. BW=Birth weight, WW=Weaning weight, SMW=Six months body weight, YBW=One yearling body weight, ADG1=Average daily gain from birth to 3 months, ADG2=Average daily gain from 3 to 12 months

#### Genetic and phenotypic correlations

Estimates of genetic correlation between BW and other studied traits ranged low to high, −0.10±0.12 between BW and ADG2, 0.59 between BW and SMW (Table-3). Similarly, WW also had moderate to high genetic correlations with other growth traits. The genetic correlation of WW with ADG1 was very high (0.99). SMW had moderate genetic correlations with other growth trait. The genetic correlation between ADG1 and ADG2 was 0.49. Moderate to the high genetic correlation of BW with body weight at subsequent ages and gain has also been reported by Gowane *et al*. [14] and Ganeshan *et al*. [25]. Estimates of genetic correlations between body weights and gain are similar with the estimates of Singh *et al*. [3]. The phenotypic correlations of BW with other body weights and gains ranged from 0.09 to 0.98. WW had high phenotypic correlation with ADG1. Estimates of phenotypic correlations among SMW, YBW, ADG1 and ADG2 were moderate except high correlation of SMW and YBW with ADG2. Estimates of phenotypic correlations among body weights and gains in this study were similar to those reported by Gowane *et al*. [14] and Momoh *et al*. [28]. The high genetic correlations between SMW with other body weights and gain suggest that the SMW can be effectively used in the selection program.

## Conclusion

The moderate to high heritability estimates for body weights at different ages and ADG is indicative of the scope of genetic improvement in these traits through selection. Keeping in view of high heritability and high positive correlations of SMW with body weights at later ages and gain, it is concluded that selection for body weights and ADG based on SMW would be the best approach for genetic improvement of the Harnali sheep for growth performance.

## Authors’ Contributions

ZSM and DSD have planned the study. L and CSP recorded the information and analyzed the data. SPD and CSP provided help in the analysis of data. L, RD and CSP drafted and revised the manuscript under the guidance of DSD and SPD. All authors read and approved the final manuscript.
